# Recent Advances in AlN-Based Acoustic Wave Resonators

**DOI:** 10.3390/mi16020205

**Published:** 2025-02-11

**Authors:** Hao Lu, Xiaorun Hao, Ling Yang, Bin Hou, Meng Zhang, Mei Wu, Jie Dong, Xiaohua Ma

**Affiliations:** School of Microelectronics, Xidian University, Xi’an 710126, China

**Keywords:** 5G, BAW, SAW, AlN, ScAlN, SMR, FBAR, HSBR, XBAW, MOCVD, PVD, ladder, lattice, reconfigurable

## Abstract

AlN-based bulk acoustic wave (BAW) filters have emerged as crucial components in 5G communication due to their high frequency, wide bandwidth, high power capacity, and compact size. This paper mainly reviews the basic principles and recent research advances of AlN-based BAW resonators, which are the backbone of BAW filters. We begin by summarizing the epitaxial growth of single-crystal, polycrystalline, and doped AlN films, with a focus on single-crystal AlN and ScAlN, which are currently the most popular. The discussion then extends to the structure and fabrication of BAW resonators, including the basic solidly mounted resonator (SMR) and the film bulk acoustic resonator (FBAR). The new Xtended Bulk Acoustic Wave (XBAW) technology is highlighted as an effective method to enhance filter bandwidth. Hybrid SAW/BAW resonators (HSBRs) combine the benefits of BAW and SAW resonators to significantly reduce temperature drift. The paper further explores the application of BAW resonators in ladder and lattice BAW filters, highlighting advancements in their design improvements. The frequency-reconfigurable BAW filter, which broadens the filter’s application range, has garnered substantial attention from researchers. Additionally, optimization algorithms for designing AlN-based BAW filters are outlined to reduce design time and improve efficiency. This work aims to serve as a reference for future research on AlN-based BAW filters and to provide insight for similar device studies.

## 1. Introduction

With the rapid development of the fifth generation of mobile communication technology (5G) worldwide, the scale of the mobile information market continues to expand; the form of service continues to change; its data transmission rate, data carrying capacity, and spectrum utilization are significantly improving; more and more functional modules are being added to wireless terminals; and the overall utilization of the frequency band continues to develop to high frequency [1]. n77 and n79 are core bands for global 5G deployments due to their wide bandwidth for higher data rates [2].

The new bands need to support up to 32% fractional bandwidth and frequencies above 7 GHz. In order to maintain device lifetime and prevent data rate degradation, RF front-end filters are required to have smaller size, higher frequency, wider bandwidth fractions, higher power handling, lower insertion loss, and lower temperature drift [3]. At present, the most mainstream application of radio frequency (RF) acoustic filters is dominated by surface acoustic wave (SAW) filters and bulk acoustic wave (BAW) filters [4]. BAW refers to acoustic waves traveling through a material’s bulk, while SAW involves waves confined to the surface. BAW filters have advantages such as high operating frequency and large power capacity compared with SAW filters, making them widely recognized. The high quality factor (Q factor) of BAW filters results in steeper skirts, while their high sound velocity and thermal conductivity give them high power-handling capabilities. Additionally, the low power consumption, high isolation, and compatibility with complementary metal oxide semiconductor (CMOS) technology, which is commonly used to construct integrated circuits, make BAW filters mainstream devices in the field of RF communication, which refers to electromagnetic wave frequencies typically ranging from 3 kHz to 300 GHz [5]. Therefore, a comprehensive understanding of the fundamental building block, the BAW resonator, is essential for designing higher-performance BAW filters.

Currently, aluminum nitride (AlN) piezoelectric films are used in almost all body acoustic wave filter devices due to their mature manufacturing process and excellent performance [6]. On the one hand, with the continuous development of Micro-Electro-Mechanical Systems (MEMS) technology, which involves small mechanical and electromechanical devices built using microfabrication techniques, the process of growing AlN thin films has gradually matured. In practice, large-sized AlN thin films can be grown quickly and stably, and their preparation is compatible with CMOS technology, making it more suitable for integrated circuits [7]. On the other hand, AlN has very stable chemical properties, a high piezoelectric coefficient, higher thermal conductivity compared to other piezoelectric materials, and a large Q value in a wide frequency range. It also has advantages such as high internal acoustic wave propagation speed. Due to its wide bandgap characteristics and high temperature resistance, AlN is being developed toward semiconductor devices that can withstand high frequencies, high powers, and high temperatures. Therefore, AlN has excellent thermal properties, chemical properties, piezoelectric performance, high mechanical strength, and high sound velocity characteristics, making it very suitable for application in acoustic devices [8,9,10].

## 2. Piezoelectric Materials and Their Epitaxial Growth Methods Used in BAW Resonators

### 2.1. Single-Crystal AlN

AlN films are classified as single-crystal AlN, poly-crystalline AlN, and doped AlN. Single-crystal AlN has higher crystal quality with fewer defects and more stable chemical properties, which is conducive to improved pressure properties and speed of sound and reduced absorption and scattering of bulk acoustic waves [11]. Single-crystal AlN has a strong polarization effect, and the heterojunction formed with GaN has a high concentration of two-dimensional electron gas, which can be applied in high-frequency and high-power fields [12]. The use of AlN significantly increases the breakdown voltage of high electron mobility transistors (HEMTs) and facilitates low-loss operation in enhanced-mode (E-mode) HEMTs. This is critical for devices that need to operate at higher voltages or in more challenging electrical environments. The use of AlN and specific treatments also enhances the overall stability and reliability of HEMTs. The potential of HEMTs with surface AlN layers for achieving high linearity and low power consumption in amplifier applications is significant [13,14,15]. The growth of AlN materials used for HEMTs can be achieved using molecular beam epitaxy (MBE) technology. The MBE process allows precise control of layer thickness, composition, and doping, making it an attractive technique for high-performance III-N transistors [16]. H. Lu et al. [17,18] found that recessed ohmic structures significantly improved the gain and output power of HEMTs, enhancing their large-signal characteristics. By rationally designing and optimizing the ohmic contact structure and passivation layer techniques, HEMTs have shown great potential in high-frequency and high-power applications.

J.B. Shealy et al. [19] grew single-crystal AlN films on SiC substrates using the metal-organic chemical vapor deposition (MOCVD) technique. Poly-crystalline AlN is usually grown using the physical vapor deposition (PVD) method. MOCVD uses metal-organic compounds to grow thin films, while PVD vaporizes solid materials to deposit thin films. Compared with poly-crystalline PVD AlN, single-crystal AlN films grown on SiC substrates using MOCVD have higher crystal quality, which is specifically manifested in (0004) X-rays. The full width at half maximum (FWHM) of the X-ray diffraction (XRD) sway curve is 0.025°. Research has shown that after thermal annealing, the FWHM of the (0002) plane of PVD-grown AlN on sapphire substrates can be reduced to as low as 147 arcseconds, which translates to less than 1.5 degrees [20]. This improvement in crystal quality has been shown to increase the piezoelectric coefficient [21]. AlN piezoelectric material has a higher longitudinal speed of sound, so a thicker material can be used at the same frequency [22] with higher thermal conductivity [23]. Y. Shen et al. [24] prepared single-crystal AlN-based BAW filters using MOCVD and poly-crystalline AlN-based BAW filters using PVD. Under the test conditions of a pulsed signal with a 1% duty cycle and an 800-μs period (applied to the middle and upper edges of the passband) and a pulsed continuous-wave signal with a 1% duty cycle and an 8-μs pulse duration for RF driving, single-crystal and polycrystalline AlN-based BAW filters were tested. The results show that the peak power capability of the single-crystal filter at the right edge of the passband is 18.1 W higher than that of the polycrystalline filter. Moreover, the insertion loss of the single-crystal filter ranges from 1.2 to 1.4 dB, which is lower than that of the polycrystalline filter (1.6–2.0 dB). Evidently, the single-crystal filter performs better in terms of power handling and insertion loss. 

**Figure 1:**
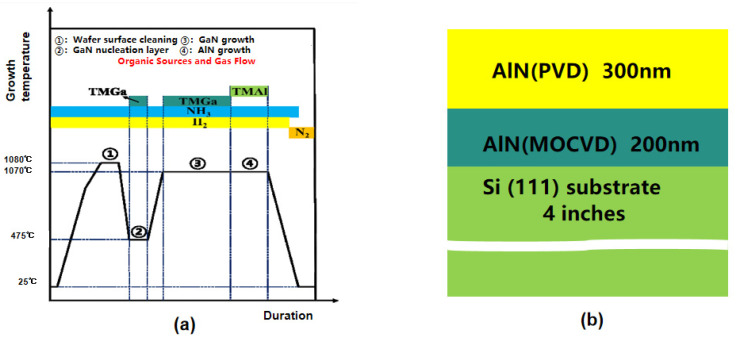
Single-crystal AlN and its epitaxial growth. (**a**) Graphical illustration of growth process under different growth temperatures [27]. Step (1) is Wafer surface cleaning; Step (2) is GaN nucleation layer; Step (3) is GaN growth; Step (4) is AlN growth. This image has been obtained with permission from IEEE Publishing. (**b**) Schematic using the epitaxial AlN layer as a sputtering template to grow the single-crystal AlN substrate. Adapted from [29].

The MOCVD method can directly grow c-axis-oriented single-crystal AIN films on sapphire substrates [25]. Currently, there is no patterned bottom electrode beneath the piezoelectric film, and integrating single-crystal film into BAW presents significant challenges. The current method involves depositing the piezoelectric film onto a substrate and then transferring it to a new substrate. Then, dry etching or wet etching is used to remove the original substrate and use the new substrate as a foundation [26]. However, this method of transferring the original substrate is challenging, costly, and time-consuming. To meet this challenge, B.H. Lin et al. [27] proposed a method for growing high-quality single-crystal AlN films by using GaN as a transition layer on a sapphire substrate. The GaN transition layer plays a crucial role by offering a template for the crystal orientation during the deposition of AlN and creating the necessary conditions for the laser lift-off process that follows. GaN with a single-crystal AlN film was detached from the sapphire substrate through laser peeling (Figure 1a).

MOCVD is often used to grow single-crystal AlN, but the internal stress is difficult to control. When the AlN film grows to 500 nm and the temperature returns to room temperature, film cracks appear and cannot be used for the preparation of BAW resonators [6]. PVD has the advantages of high film formation rate, low substrate temperature, dense film layer, low internal stress, strong adhesion, and good compatibility with the CMOS process [28]. However, the quality of AlN crystal using magnetron sputtering is poor, and there are many grain boundaries and defects. In order to combine the advantages of both methods, R.D. Qin et al. [29] proposed the use of two-step deposition to prepare single-crystal AlN. First, 200 nm thick AIN films were grown epitaxically on a 4-inch Si (111) substrate using the MOCVD method, and then the single-crystal AIN template was transferred to the PVD system, where an additional 300 nm AIN film was deposited (Figure 1b). For the (0002) peak, MOCVD-grown AlN has an FWHM of 0.45, MOCVD+PVD-grown AlN has an FWHM of 0.47, and PVD-grown AlN has a significantly wider FWHM of 2.13. The results show that the two-step growth method combines the advantages of MOVCD and PVD to effectively regulate the stress of single-crystal AlN films, which has broad application prospects.

### 2.2. Doped AlN

Since the effective electromechanical coupling coefficient ($k_{eff}^{2}$) of the piezoelectric layer determines the relative bandwidth of the BAW filter [30], a way must be found to improve $k_{eff}^{2}$ in order to meet the requirements of RF front-end filters. Numerous studies have shown that doping certain elements in AlN changes its piezoelectric properties and $k_{eff}^{2}$. Doping can be divided into single doping and co-doping. At present, the single doping elements studied include Sc [31,32,33,34,35,36], Ti [37,38], Ta [39], V [39], Mg [40], Er [41], Y [42], Cr [31], etc. Among them, doping with Ti, V, Mg, and other elements will reduce the piezoelectric properties of AlN. Co-doping mainly consists of Mg and other elements, including Mg + Zr [43], Mg + Hf [44], Mg + Ti [45], Mg + Nb [46,47], etc. Controlling the doping ratio of Mg and Zr/Hf/Ti/Nb and other elements will also significantly improve the piezoelectric properties of AlN.

**Figure 2:**
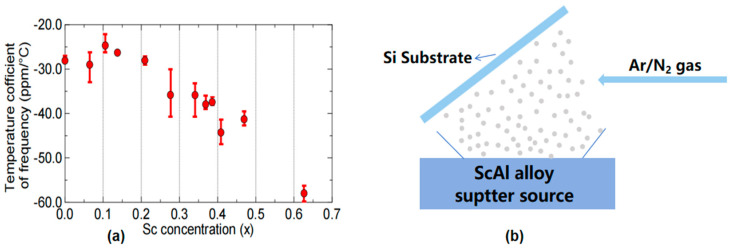
Properties and preparation of doped AlN. (**a**) TCF of Sc_x_Al_1−x_N films as a function of Sc concentration (x) [30]. This image has been obtained with permission from IEEE Publishing. (**b**) Method for depositing c-axis zig-zag ScAlN multilayers. Adapted from [56].

M. Akiyama et al. [48] first found that doping of Sc in AlN could greatly improve the piezoelectric characteristics of AlN, and the d33 (the piezoelectric charge coefficient representing the polarization generated in the direction of an applied mechanical stress) of the piezoelectric film increased by 400%. As a kind of nitride semiconductor, ScAlN is still a hot research topic due to its improved piezoelectric performance compared to pure AlN. Structurally, the incorporation of Sc into the AlN lattice causes slight lattice distortion, leading to changes in both mechanical and electrical properties. The FWHM of the recently studied ScAlN can reach 1.9 deg [49,50], indicating a high crystalline quality and uniformity, which is beneficial for improving the material’s piezoelectric properties and overall device performance. This lattice expansion, due to the larger ionic radius of Sc compared to Al, has been shown to affect the crystallinity and other key material properties, which are critical in BAW resonator performance. For comparison, epitaxial AlN typically retains a highly crystalline wurtzite structure with minimal defects, while Sc doping can introduce strain, affecting the overall crystal quality [51,52,53,54,55,56]. M. Moreira et al. [51] prepared BAW (FBAR) based on three kinds of ScAlN with different Sc contents. The results showed that when the Sc content was 3%, 9%, and 15%, the $k_{eff}^{2}$ showed an upward trend, which was 7.55%, 7.55%, and 12%, respectively. However, the quality factor (Q) showed a downward trend, which was 601, 513, and 348, respectively. With the improvement of $k_{eff}^{2}$, however, doping with Sc causes a serious problem: the deterioration of the mechanical quality factor (Q_m_) [52]. Furthermore, temperature coefficients of frequency (TCF) degradation may occur in ScAlN films compared to AlN because the elastic constants of Sc_x_Al_1−x_N films decrease with increasing Sc concentration [53]. H. Igeta et al. [30] investigated the effect of Sc doping concentration (Sc_x_Al_1−x_N) on TCF. When x < 0.2, there was no significant change in TCF, and when x > 0.2, TCF deteriorated. Therefore films in the region of x > 0.2 are promising for film resonator application (Figure 2a). Under their experimental conditions, the TCF values of ScAlN and SiO_2_ exhibited opposite signs. To address this discrepancy, SiO_2_ was introduced, and its properties were carefully controlled to mitigate the deterioration of TCF. Although it achieves near-zero TCF in the piezoelectric layer, it experiences a reduction in the $k^{2}$ value due to the non-piezoelectric nature of SiO_2_. In [30] the value of $k^{2}$ is only 7.9%. Similarly, K. Izumi et al. [54] also used alternating piezoelectric and non-piezoelectric layers to fabricate BAW resonators which can be fabricated in a large area with common sputtering systems (ScAlN and SiO_2_ were alternately arranged in the report).

ScAlN/SiO_2_ films can be prepared by RF magnetron sputtering [54]. First, the c-axis-oriented ScAlN film is grown on the bottom electrode film/silica glass substrate by RF magnetron sputtering, and then the SiO_2_ film is deposited using the SiO_2_ target. The process is then repeated. In addition to ScAlN films with normal c-axis orientation, c-axis-tilted ScAlN films can be prepared in some scenarios. As shown in Figure 2b, c-axis-tilted films and c-axis-normal films were fabricated using single and dual RF magnetron sputtering systems, respectively [55]. Examples of preparing c-axis-tilted ScAlN films are reported in the literature [33,56]. Using glancing angle magnetron sputtering, ScAlN films with a c-axis tilt were deposited on a silica glass substrate with a bottom electrode film. The substrate was positioned at 60° from the target surface to achieve a c-axis tilt of approximately 50° [56]. The target surface was ScAl metal alloy (Sc_43_Al_57_) (Figure 2b).

## 3. The Structure and Fabrication Process of AlN-Based BAW Resonators

### 3.1. Classification

**Figure 3:**
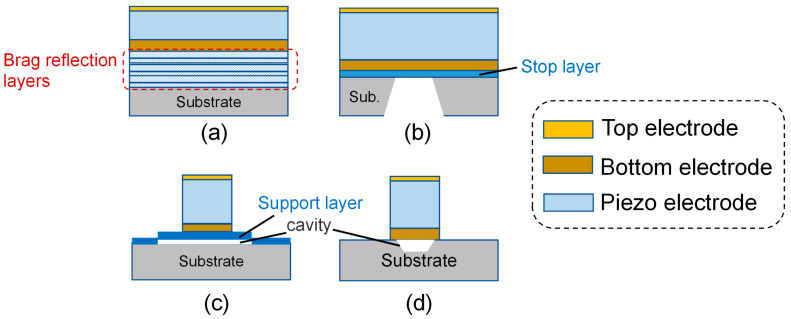
The structure of BAW. (**a**–**d**) SMR, back silicon etching-type FBAR, lower concave cavity-type FBAR, and upper convex cavity-type FBAR, respectively.

BAW resonators can be divided into two categories: one is based on film bulk acoustic wave resonator (FBAR) resonators, and the other is based on solid-state solidly mounted resonator (SMR) resonators (Figure 3a). According to the different reflection methods, BAW resonators with the FBAR structure are divided into back silicon etching-type FBAR, lower concave cavity-type FBAR, and upper convex cavity-type FBAR [57] (Figure 3b–d). SMR resonators achieve acoustic reflection through the air interface between the solid medium and the upper and lower surfaces, or Bragg reflection layer mutation. Compared with SMR, FBAR has a higher quality factor (Q) and a higher $k_{eff}^{2}$ [58]. With the development of MEMS, the FBAR filter has the advantages of low power consumption, high power capacity, and it can be combined with CMOS. It has become a research hotspot for 5G RF filters [59]. SMR uses the Bragg reflection layer to limit the sound wave within the piezoelectric oscillation stack. The Bragg reflection layer generally uses W and SiO_2_ as the acoustic layer with high and low impedance, because the acoustic impedance difference between W and SiO_2_ is large, which can effectively restrain the sound wave [6]. The biggest advantages of SMR are its strong mechanical fastness, good integration, and not needing to use MEMS technology. The disadvantages of SMR are that multi-layer films need to be prepared, the process cost is higher than that of air gap FBAR, and the acoustic reflection effect of the Bragg reflection layer is not as good as that of air, so the Q value of SMR is generally lower than that of air gap FBAR [60].

### 3.2. Principle and Characterization

The operational principle of BAW resonators can be succinctly described as follows [61]: Upon application of a specific RF voltage frequency across the upper and lower electrodes, the piezoelectric material within the device undergoes an inverse piezoelectric effect within its core, inducing mechanical vibrations. These vibrations then convert the RF electrical signal into an acoustic wave that travels through the thickness of the piezoelectric layer. Given that the upper and lower surfaces of the resonant oscillation region are exposed to air, which serves as an effective acoustic reflector, the acoustic wave experiences total reflection. Consequently, the wave undergoes multiple reflections between the two air interfaces, resulting in the formation of an acoustic standing wave within the resonant oscillation region, thereby achieving resonance.

The performance evaluation of BAW resonators typically revolves around two key parameters [6,59]: the effective piezoelectric coupling coefficient ($k_{eff}^{2}$) and the quality factor (Q). $k_{eff}^{2}$ characterizes the ratio of energy conversion between the mechanical and electrical domains, thereby determining the bandwidth of BAW filters. Generally, a larger $k_{eff}^{2}$ can meet the wide bandwidth requirements of RF filters in 5G communication systems. Research suggests that $k_{eff}^{2}$ for advanced materials like AlScN can reach 12–16%. The Q of a BAW resonator measures how efficiently it stores energy versus losing it to heat. A higher Q indicates sharper resonance, meaning the filter can better separate closely spaced frequencies with less energy loss. It directly impacts the filter’s selectivity and performance. The Q factor typically ranges from 700 to 1000, depending on the operating frequency and material used. Thus, $k_{eff}^{2}$ × Q is called the figure of merit (FOM) [62]. In advanced BAW resonators using materials like AlScN, the FOM often reaches 60 to 160 for filters operating in the 2~5 GHz range. Research indicates that the optimal value for $k_{eff}^{2}$ is twice the relative bandwidth of designed BAW filters [60].

### 3.3. The Design and Fabrication of BAW Resonators

#### 3.3.1. BAW-SMR Resonator

**Figure 4:**
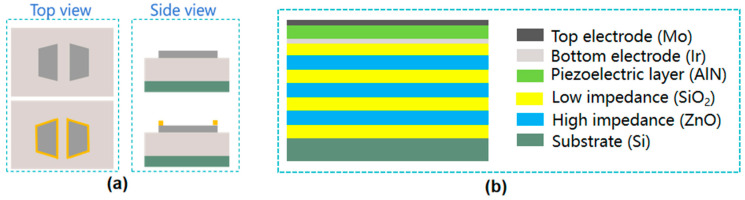
Fabrication of BAW-SMR. (**a**) Schematic representation of SMR structures without and with electrode frames. Adapted from [66]. (**b**) Schematic diagram of SMR on acoustic reflector diagram. The bottom electrode cannot be directly accessed by the RF probe and is instead excited by the capacitive coupling effect. Adapted from [64].

The BAW-SMR exhibits robust mechanical strength and excellent integration characteristics. And there is a heat conduction path leading to the substrate in the structure, which can well dissipate heat through the substrate [63], so it has an excellent TCF [64]. It has many application scenarios in communication systems. D. Mercier et al. [65] fabricated filters for space using BAW resonators with the SMR structure. The resonators and filters were manufactured on 200 mm high-resistivity silicon wafers with a resistivity greater than 2 kΩ·cm. The resonator structure comprised an AlN piezoelectric layer sandwiched between Mo electrodes, situated atop a bragg reflector. This reflector, composed of W and SiO_2_, served to confine the acoustic wave effectively. Additionally, a silicon nitride (Si_3_N_4_) passivation layer was uniformly deposited over the resonator to enhance reliability. It was selectively etched to establish electrical connections with the Mo electrodes. The RF transmission lines and electrode contacts were fabricated using Al. The resonance frequency of the acoustic resonators was finely tuned by partially etching the Si_3_N_4_ layer atop each resonator. Figure 3a shows a basic cross-section schematic of SMR technology. Based on the above, Andreas Bogner et al. [66] used two-layer Al/W electrodes to optimize the effective coupling of W while maintaining low electrical losses of Al. As shown in Figure 6b in reference [66], the extracted effective $k_{eff}^{2}$ showed lower values around 12.5%, which could be attributed to the usually lower quality at the beginning of crystal growth of the used thin films. The inhomogeneous distribution of Q is mainly determined by mirror performance bias. Literature [66] also compares the situations with and without electrode frames (Figure 4a). In the research of n41 band filters, for suppressing spurious modes, electrode apodization was adopted in some cases, and electrode frames were only applied to n41 single resonators. The results show that the insertion loss increases when there is no electrode frame. Moreover, in some samples, additional notches appear due to the inhomogeneity of the mechanical polishing process of the bottom electrode, while this phenomenon was not observed in the ladder experiment of the sample with electrode frames.

SiO_2_ in the Bragg reflection layer is the most common low-impedance material due to its appropriate acoustic properties in SMR-BAW. Metallic materials such as W [65,67], Ir [68], and Mo [69,70] are usually the ones occupying these positions in high-impedance materials. However, undesired capacitive coupling may occur if specific electrode grounding methods are used [67]. To avoid the aforementioned interference, accessing the bottom electrode by capacitive coupling avoids the use of additional masking steps and etching through the AlN piezoelectric layer [64] (Figure 4b).

Since the operating frequency of BAW-SMR is completely determined by the set of layers, especially their thickness, it is crucial to carefully control these parameters. This means that a set of resonators operating at a specific frequency can be fabricated at a time. However, it is hardly possible to synthesize two filters for different standards [71]. This will increase the complexity of the manufacturing process. In order to solve this problem, it is proposed to deposit piezoelectric films with different thicknesses on the same substrate [72]. At present, there are two realization ideas. The first method is to deposit the thinner film first for the highest frequency filter, and then continue to deposit the thicker film at the location of the low-frequency filter [73]. However, the piezoelectric properties of thicker films will be decreased [71]. The second method involves depositing the thickest film first and then using etching to obtain the required film thickness for the RF filter. This method will not reduce the piezoelectric properties of the membrane but will increase the roughness of the membrane surface, which may reduce the electromechanical coupling coefficient. In order to solve the above shortcomings, E. Iborra et al. [74] proposed a new method based on multiple deposition and imaging of piezoelectric layers.

#### 3.3.2. BAW-FBAR Resonator

**Figure 5:**
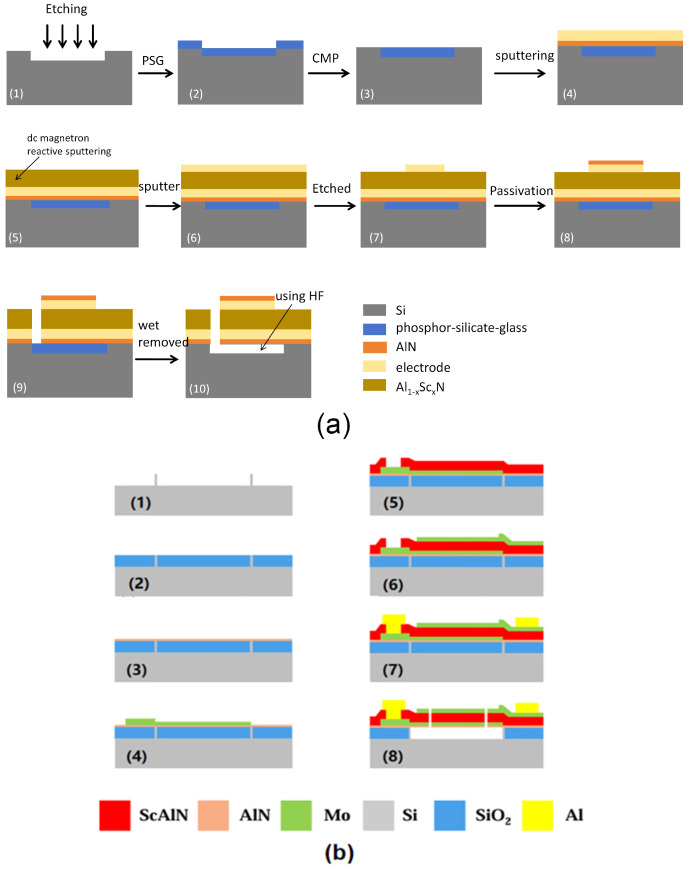

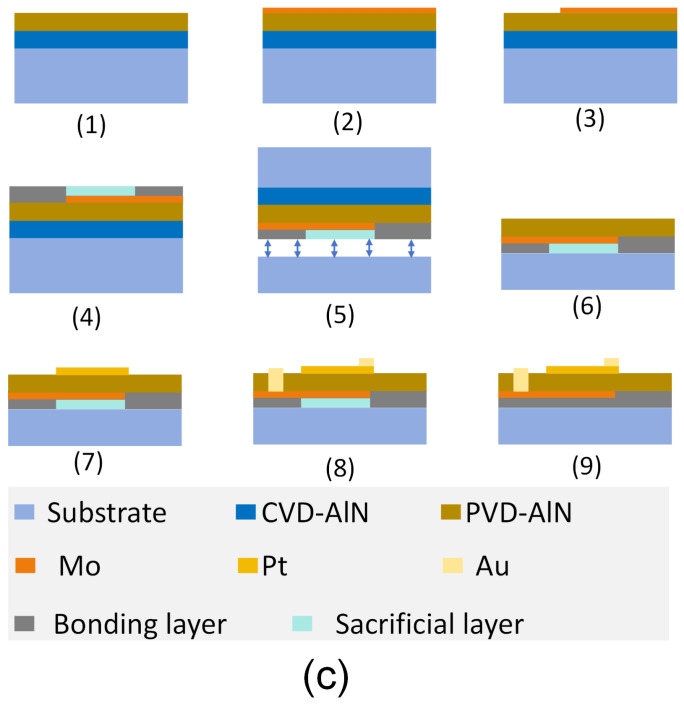
The fabrication of BAW-FBAR. (**a**) Fabrication process of the FBAR device. Adapted from [75]. (**b**) Fabrication of AlScN-based upper mounted cavity-type FBAR. Adapted from [82] (**c**) Wafer bonding and layer transfer techniques. Adapted from [29].

The key difference between BAW-FBAR and BAW-SMR is that the FBAR resonator forms a cavity under the bottom electrode. R. Ding et al. [75] reported a conventional method for fabricating FBAR resonators. First, a 4 µm cavity was etched on the silicon wafer. Then, 5 µm phosphor-silicon glass (PSG) was deposited on the silicon wafer for chemical mechanical polishing (CMP) flat polishing. To improve the crystal axis orientation of the bottom electrodes [76], a 30 nm AlN seed layer was deposited on the silicon substrate and 150 nm Mo bottom electrodes were sputtered randomly. The AlScN piezoelectric films were then epitaxially grown by DC magnetron sputtering. Subsequently, a top Mo film with a thickness of 150 nm was deposited by dc sputtering, then patterned and etched using inductively coupled plasma (ICP). A 100 nm thick AlN passivation layer was also deposited in order to protect Mo from oxidation [77]. This layer also served to fine-tune the resonant frequency. Finally, in order to release the sacrificial layer of PSG to obtain the cavity, the PSG layer was reached through a via hole [27] and hydrogen fluoride (HF) was used (Figure 5a).

The cavity can also be formed on top of the Si substrate, and the preparation process is different from the process in which the cavity is embedded in the Si substrate. Y. Zou et al. [78] fabricated FBAR resonators by forming cavities on Si substrates. Different from the above methods, Y. Zou et al. [79,80,81] first used high-resistance silicon etching to form an isolation wall to define the shape of the cavity, which was then filled with SiO_2_ as the support for the growth of the upper layer material, which was later removed to release the cavity. Two layers of Mo were deposited on the top, with 100 nm thick Mo used as the top electrode and 37 nm thick Mo used as the mass loading layer. After that, 1 µm thick Al was deposited by magnetron sputtering and patterned to define the probing pad. Figure 5b illustrates the flow of the preparation [82].

R.D. Qin et al. [29] introduced wafer bonding and layer transfer methods for inserting the bottom electrode utilizing single-crystal AlN films epitaxially grown using a two-step growth method. A 200 nm thick Mo was first deposited on the AlN surface by sputtering as the bottom electrode. Then, a sacrificial layer was deposited and flattened. The piezoelectric film was then stacked onto another highly resistive silicon using a wafer bonding and substrate removal process. Pt electrodes, AlN-VIAs, and Ti/Au substrates were processed on them. Finally, the sacrificial layer was selectively removed to create a cavity beneath the resonance region (Figure 5c).

#### 3.3.3. BAW-XBAW Resonator

Conventional BAW resonator technology is limited in its ability to address wider bandwidth requirements owing to its relatively modest piezoelectric coupling. Consequently, there has been considerable interest in a novel device known as Xtended Bulk Acoustic Wave (XBAW), which has garnered significant attention [83,84,85,86]. XBAW is a new process for manufacturing BAW resonators, offering optimized performance over traditional designs. While both XBAW and FBAR resonators use bulk acoustic waves, XBAW resonators are not classified as FBAR resonators but are part of advanced BAW resonators technology. Thus, BAW-XBAW resonators are a specific type of BAW device. The XBAW process has the advantage of being compatible with both single-crystal and poly-crystalline AlN piezoelectric materials [87]. XBAW resonators feature dual air interfaces [24] and a unique transferred substrate manufacturing process, which can utilize AlScN material with high Sc concentrations, deposited via either PVD or MOCVD. The XBAW process, in contrast to traditional BAW resonator fabrication methods, lies in the uniformity and continuity of the produced piezoelectric film. In employing the XBAW technique, the resultant film maintains a flat, uninterrupted surface, characterized by a consistent grain structure and high-quality acoustic properties across the entire device, from which a high FOM resonator will be fabricated [88,89]. The transfer process in XBAW eliminates the need for sputtering the piezoelectric film over a bottom electrode, enabling the growth on any arbitrary and high-quality substrate. Thus, the XBAW process also enables removal of that portion of the piezoelectric film that incorporates a higher defect level [90,91].

**Figure 6:**
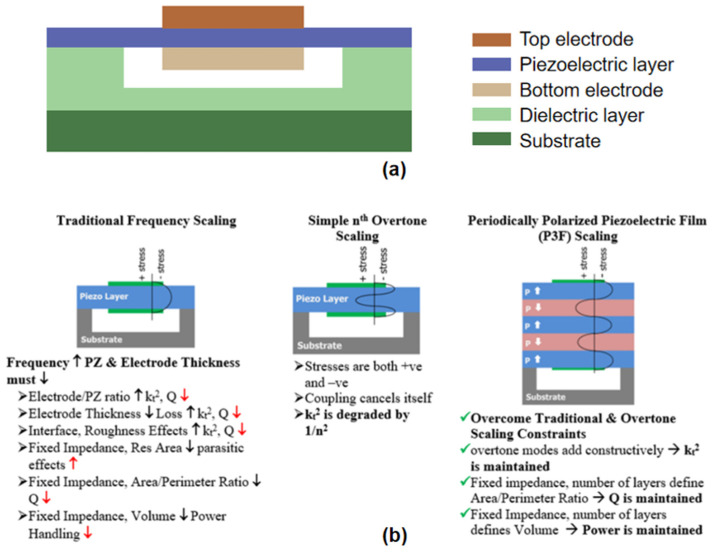
BAW-XBAW resonator. (**a**) Simplified section view of the XBAR resonator. Adapted from [92]. (**b**) Traditional frequency scaling, simple nth overtone scaling and P3F scaling [89]. This image has been obtained with permission from IEEE Publishing.

Y. Shen et al. [92] introduced the manufacturing process of XBAW: Firstly, a doped AlN piezoelectric layer with a thickness of 0.42 μm was grown on a Si substrate with a diameter of 150 mm. After sputtering and deposition of the electrode and a two-sided wafer process (an 11-mask layer, two-sided wafer process), resonators with two air interfaces were generated. The backside resonator electrode was routed to the topside of the wafer using vias in the doped AlN thin film [88]. Figure 6a shows a schematic diagram of the XBAW resonator. Since XBAW technology has the advantage of combining multilayer piezoelectric films at the same time, such as single-crystal ScAlN and poly-crystalline ScAlN. Thus, a new technique has been developed to enhance FOM: a fabricable, periodically polarized piezoelectric film (P3F) BAW resonator based on the XBAW process [89,93]. P3F is constructed by stacking oppositely polarized piezoelectric layers on top of each other, which increases the total piezoelectric thickness and avoids any decrease in electromechanical coupling. The advantages of P3F are shown in Figure 6b. P3F enhances acoustic resonator performance by increasing the effective piezoelectric thickness, improving acoustic energy confinement, and maintaining electromechanical coupling. This approach provides frequency scaling benefits for millimeter wave applications while reducing filter bandwidth loss. Overall, P3F significantly boosts the acoustic Q factor and mitigates resistive losses, improving resonator efficacy.

The currently studied XBAW resonators are summarized in Table 1. The filters using this process will have great application scenarios in the future 5G/6G communication field because of their excellent performance.

#### 3.3.4. Hybrid SAW/BAW Resonators (HSBRs)

**Figure 7:**
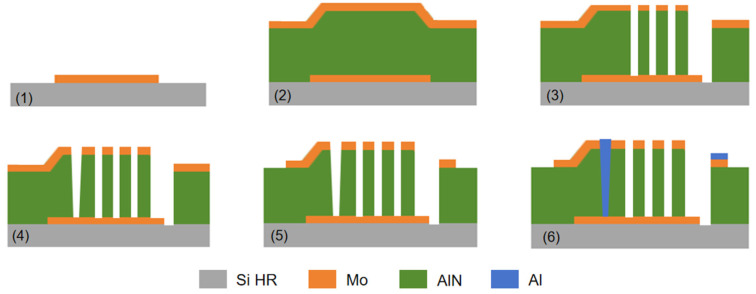
Construction and fabrication of HSBRs. Six-mask process flow for fabricating HSBRs. Adapted from [95].

In HSBRs, which are proposed by V. Plessky et al. [94], BAW resonators replace the fingers of the cross transducer (IDT) of each SAW resonator, which is periodically repeated through the SAW wavelength. The BAW resonator vibrates periodically on the substrate, generating surface acoustic waves in the substrate [90]. The efficient transduction of SAW by vibrating BAW resonators depends upon the fact that both waves have the same wavelength (λ_SAW_ = λ_BAW_), which means that the thickness of the piezoelectric thin-film (AlN) should be λ_SAW_/2. The literature [95] gives a general preparation process based on a six-mask process (Figure 7): The common bottom Mo electrode for all transducers is deposited first and then patterned. AIN piezoelectric films, Mo electrodes, and SiO_2_ hard masks are fabricated on this basis. UV lithography and dry etching are then used to define the transducer separation grooves. After chemical removal of the etching mask, the Mo and AlN films are patterned again using dry and wet etching methods, respectively, to expose the electrical contacts to the bottom electrode. The top Mo electrode is then shaped via dry etching, and Al contact pads are added using the lift-off process to facilitate electrical probing. Test results show that HSBRs can achieve high Q values. The electromechanical coupling factors of the pseudo-BAW mode can reach up to 2%, and its quality factors can reach as high as 1900.

**Figure 8:**
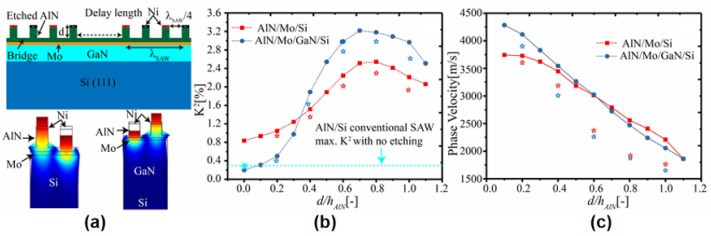
Improvement of HSBR performance. (**a**) Schematic of the coupled BAW/SAW resonator proposed in [101]. (**b**) The estimation results by FEM (marked stars show the experimental results and phase velocity of the excited mode for Si and GaN substrates) [101]. (**c**) The phase velocity of the excited mode for Si and GaN substrates [101]. Images (**a**–**c**) have been obtained with permission from IEEE Publishing.

The electromechanical coupling coefficient ($k^{2}$) and phase velocity (v) are critical parameters for SAW devices [96,97]. However, the $k^{2}$ value of HSBRs is not ideal [98]. S. Barsoum et al. [99,100] studied the effect of the transducer aspect ratio on the electromechanical coupling factor. The maximum $k^{2}$ value of pseudo-SAW mode is only 2.4%, and the maximum $k^{2}$ value of pseudo-BAW mode is 3.4%, which is significantly lower than that of BAW resonators. In order to enhance $k^{2}$, one method is to deposit a low-impedance layer on the Si substrate, then deposit the bottom electrode, and then deposit a high-impedance layer to reduce the acoustic loss. Finally, the top BAW resonator part is fabricated [35,101] (Figure 8a). The structure proposed in [35] is referred to as HAL, where the high-impedance material is AlN, the low-impedance material is SiO_2_, and the piezoelectric layer is ScAlN. As shown in Figure 4 in [35], by optimization of the structure configuration parameters, the obtained $k^{2}$ has a remarkable value of 21%, where the v is about 5100 m/s. The study [101] investigated the characteristics of coupled BAW/SAW devices under different conditions. When the ratio of the etching depth of the interdigitated transducer (IDT) fingers to the total AlN thickness was varied, the $k^{2}$ of the coupled BAW/SAW devices on non-piezoelectric substrates was significantly enhanced compared with that of the conventional AlN/Si-based SAW devices. When a GaN piezoelectric substrate was used, the coupling efficiency was also greatly improved. Meanwhile, the phase velocities of both piezoelectric and non-piezoelectric substrates decreased rapidly as the above-mentioned ratio increased, which was due to the fact that a BAW mechanical source was adopted to excite SAW in these devices. Finite-element method (FEM) analysis showed that the high elastic constant of GaN was the reason for the higher $k^{2}$ in AlN/Mo/GaN devices compared with AlN/Mo/Si devices (Figure 8b,c). Y. Zhang et al. [102] provided a new idea in which 6H-SiC was used as the substrate and the piezoelectric layer was ScAlN, with SiO_2_ filling the grooves. It had a remarkable $k_{eff}^{2}$ value of 14.55% and a high v above 7500 m/s. Furthermore, acoustic metamaterial devices also have significant potential to achieve high $k^{2}$. X. Y. Zhao et al. [49,103] provided complementary approaches to enhance $k^{2}$ using acoustic metamaterials. The studies introduced AlN-based two-dimensional resonant rods (2DRRs) with a $k^{2}$ value of 7.4%, and a record $k^{2}$ value of 23.9% was achieved using AlScN. The enhanced $k^{2}$ allows for ultra-wideband filters suitable for next-generation 5G and 6G applications.

## 4. The Application of AlN-Based BAW Resonators

AlN-based BAW resonators are primarily used to construct BAW filters. Therefore, this chapter focuses on reviewing the current research directions from the perspective of BAW filters, including circuit topologies, methods for expanding BAW filter bandwidth, frequency reconfiguration technologies, and studies aimed at improving the speed of circuit design.

**Figure 9:**
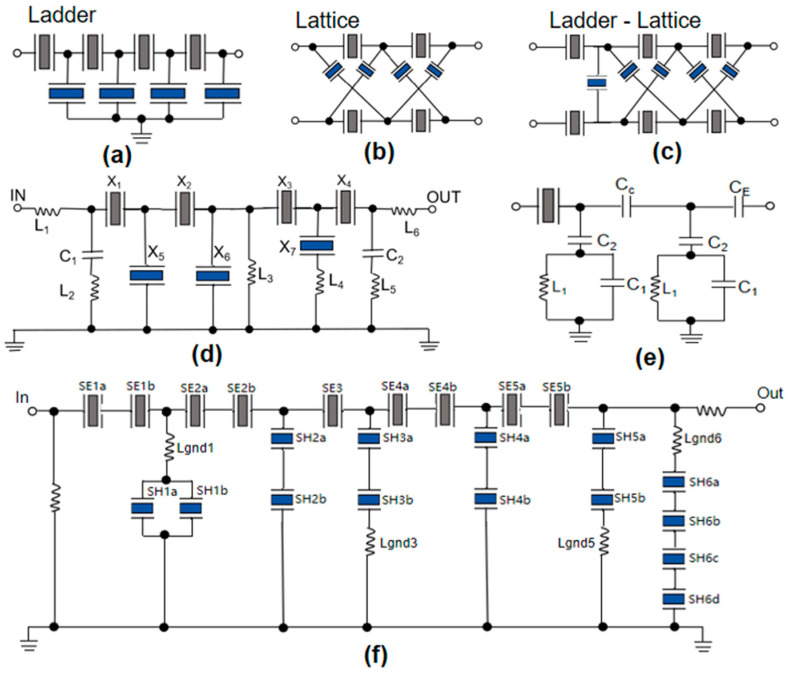
The measures to expand bandwidth. (**a**) Ladder filter. (**b**) lattice filter. (**c**) ladder-lattice filter [78]. (**d**) Topological structure of the ladder-type FBAR filter circuit. Adapted from [109]. (**e**) The filter topology structure combining BAW and IPD technologies. Adapted from [110] (**f**) Topology of the hybrid B41 BAW filter. Adapted from [111] with AlN resonators.

The basic BAW filter chip structure includes the ladder structure and lattice structure [104]. The individual resonators in these two topologies are not coupled and work separately, and their structures are shown in Figure 9a–c. The ladder structure usually grounds the parallel resonators to realize the single-ended input and output structure, which is widely used. The structure of the ladder connection topology is mainly composed of series resonators and parallel resonators, and the resonant frequency of series resonators is always higher than that of parallel resonators [105]. The ladder structure has transmission zero out of band, good proximal suppression but poor distal suppression, it directly processes single-ended signals, does not need to introduce additional devices, and it is conducive to miniaturization [75]. The lattice connection structure is a double-terminal output structure, which can achieve wider bandwidth and better remote rejection, but it has the disadvantage of a poor rectangular coefficient. Moreover, this kind of filter needs to introduce additional devices to process the input signal, which will increase the volume of the filter and is not conducive to integrated processing, so the applications of this structure are limited [106]. In order to combine the advantages of both structures, it is common to mix the two structures [107,108]. The new structure not only maintains the broadband property of the lattice structure but also introduces transmission zero points through the ladder structure, which makes the filter have steep edge characteristics.

In order to enhance the bandwidth of BAW filters, several effective approaches are available. On the basis of the ladder structure, Q. Wang et al. [109] found that when the FBAR resonator was connected in series or parallel with the inductor element, the spacing between the series and parallel resonant frequencies of the resonators could be increased, thus increasing the electromechanical coupling coefficient of the resonators to a certain extent, which was conducive to the realization of wideband filter design. The topological structure is shown in Figure 9d. The relative bandwidth of 1 dB was up to 8.7%. Y. Zou et al. [78] formed Mo layers on the connecting bands of each FBAR resonator. It not only expanded the bandwidth but also improved the notch formed in the passband of the traditional filter. The measured voltage standing wave ratio (VSWR) of the modified FBAR filter is below 2 in the pass-band, so it demonstrates a favorable match to 50Ω without the need for any auxiliary circuits. In general, the filter exhibits a low insertion loss of 1.804 dB, a broad bandwidth of 189 MHz, and an out-of-band rejection of 30 dB. Y. Jang et al. [110] innovatively combined BAW resonators and IPD filters (Figure 9e), demonstrating superior attenuation performance and boasting an impressive 900 MHz wide passband. Also using the idea of “hybridization”, E. Guerrero et al. [111] designed a new BAW filter based on the ladder configuration by combining AlN-based BAW resonators and ScAlN-based BAW resonators, called a hybrid BAW filter (Figure 9f), which not only improved the bandwidth but also improved the skirt steepness of the filter. M. Z. Koohi et al. [112] introduced a cascaded FBAR (cas-FBAR) constructed on an oxide/silicon substrate using a fully epitaxial metal/Al_0.8_Sc_0.2_N/metal layer. This cas-FBAR achieved the highest FOM of 14.71 at 19.11 GHz. The electromechanical coupling coefficient reached 10.14%, which is the highest value reported in k-band FBAR devices so far.

In order to broaden the application range of BAW filters, it is necessary to study frequency reconfigurable technology. The AlN-based BAW filter can change the thickness of the piezoelectric layer by applying a large external DC electric field and then achieve frequency tuning, but the achieved tunability is very limited, usually less than or much less than 1% [113]. The electrothermal technique, which utilizes a micro heater to change the resonator’s frequency, shows a large tuning capability (4500 to 96,800 ppm), but its high power requirement (2.8 mW) limits its application [114,115,116]. W. Pang et al. [117] utilized an electrostatic actuator to reconstruct the frequency, which achieved a tuning range of 7826 to 15,000 ppm. B. Kim et al. [118] proposed a new approach to tune the resonant frequencies in overtone resonators using reactive components such as capacitors and inductors. The tuning range achieved by the reported resonator was 1500 ppm. Along the same lines, Y. Izhar et al. [119] improved the tunability range (17,783~24,694 ppm) by using ScAlN materials and the novel XBAW process. BAW filters using ferroelectric materials (such as BST) as piezoelectric layers have relatively good reconfigurable properties [120,121,122]. Recent studies have shown that ScAlN also has excellent ferroelectric properties [123]. Therefore, tunable BAW resonators based on ScAlN, with frequencies reaching up to 10 GHz, continue to be developed [124,125]. Along these lines, D. Mo et al. [126,127] alternately stacked N ferroelectric ScAlN films and N + 1 metal electrodes and realized the independent polarization switching of each piezoelectric film to achieve the switching of the center frequency. In addition to the discussed technologies, Lamb wave resonators (LWR), leveraging their distinctive vibration modes and structural design, enable the tuning of resonant frequencies by modifying material thickness or electrode configurations. This tunability facilitates the development of multi-mode or dual-passband filters, which are well-suited to meet the dynamic frequency reconfiguration requirements of modern communication systems, such as those in 5G networks [128,129].

Given the significant increase in the demand for filters, it is significant to enhance the speed of filter circuit design. Filter design usually uses ADS to build models and simulations. When using ADS for simulations and optimization, it is very complex to adjust the structural parameters of the BAW filters according to the design objectives [130]. When optimizing, the optimization function will automatically search for parameters to reach the target, but it takes a long time when the optimization range is wide. In order to improve the speed and accuracy of design, J. L. An et al. [131] proposed a mode for optimizing FBAR parameters based on deep learning. This model gave appropriate initial values for the 12 design parameters of FBAR and narrowed the optimization range of each parameter, thus the design time of FBAR filters was shortened in ADS simulations. H. Zhu et al. [132] combined the Mason model with the fine multi-physical coupling model based on the space mapping optimization algorithm. This approach enhanced design efficiency by ensuring consistency between coarse and fine models, significantly reducing computational time. The Mason model [132,133] is an equivalent circuit model that transforms mechanical behavior into electrical behavior, using parameters such as capacitance, inductance, and resistance to describe dynamic characteristics and predict performance metrics like resonant frequency and electromechanical coupling coefficients. By contrast, the fine multi-physical coupling model [134,135] analyzes the interactions of multiple physical fields, integrating electrical, mechanical, and acoustic coupling. It employs high-precision numerical methods, such as finite element analysis, to achieve comprehensive modeling and optimization of system behavior. Together, these models provide effective tools for analysis and optimization in acoustic device design. In order to develop an accurate and effective tool for FBAR coupled vibration analysis, 2D plate theory was derived and established by N. Li et al. [136] based on the frame-like structure. By simplifying the plate structure to a two-dimensional model, this theory focuses on in-plane deformation and stress, effectively ignoring the thickness direction. And the accuracy and efficiency of the 2D plate theory were ensured by comparison with the finite element method. This method can well suppress the influence of other coupling modes on the filter performance.

## 5. Conclusions

This article provides a comprehensive review of AlN-based BAW resonators, the essential components of BAW filters, which play a critical role in 5G communication systems. This paper includes the epitaxial growth of piezoelectric films, the structure and fabrication of BAW resonators, and the application of BAW resonators in BAW filters. Compared with traditional AlN, doped AlN (especially ScAlN) has better performance. The new XBAW technology provides a new idea for expanding the bandwidth of BAW filters. In addition, frequency reconfiguration as a filter technology provides broader application scenarios for BAW filters. The insights gained from this extensive examination underscore the immense potential of AlN-based BAW resonators to revolutionize the field of telecommunications.

## Figures and Tables

**Table 1:** The characteristic of BAW-XBAR resonators.

Ref.	Piezo Layer	Center Freq. (GHz)	$k_{eff}^{2}$ (%)	Q	$\mathrm{FOM}\;(k_{eff}^{2}$ × Q)
[88]	Al_0.72_Sc_0.28_N	3.5	16.7	951	158
[89]	Al_0.2_Sc_0.2_N on Al_0.2_Sc_0.2_N (P3F)	10.7	7.94	342	27.2
[89]	Al_0.3_Sc_0.2_N on Al_0.2_Sc_0.2_N (P3F)	18.4	7.55	260	20
[90]	highly doped AlScN	6.4	20	500	100
[92]	Doped AlN	5.66	10.24	1479	151
[93]	Single and polycrystalline AlScN (P3F)	8.76	13	935	122
[93]	Single and polycrystalline AlScN (P3F)	10.72	10	789	79

## Data Availability

No new data were created or analyzed in this study.
